# Assessment of Cu applications in two contrasting soils—effects on soil microbial activity and the fungal community structure

**DOI:** 10.1007/s10646-017-1888-y

**Published:** 2018-01-02

**Authors:** Katharina M. Keiblinger, Martin Schneider, Markus Gorfer, Melanie Paumann, Evi Deltedesco, Harald Berger, Lisa Jöchlinger, Axel Mentler, Sophie Zechmeister-Boltenstern, Gerhard Soja, Franz Zehetner

**Affiliations:** 1 Institute of Soil Research, University of Natural Resources and Life Sciences, Vienna, Austria; 2 AIT Austrian Institute of Technology, Center for Energy, Business Unit Environmental Resources & Technologies, Tulln, Austria; 3 AIT Austrian Institute of Technology, Center for Health and Bioresources, Business Unit Bioresources, Tulln, Austria; 4 Symbiocyte Berger, Tulln, Austria

**Keywords:** Soil microbial activity, Soil fungal community, Ecotoxicity, ITS region

## Abstract

Copper (Cu)-based fungicides have been used in viticulture to prevent downy mildew since the end of the 19th century, and are still used today to reduce fungal diseases. Consequently, Cu has built up in many vineyard soils, and it is still unclear how this affects soil functioning. The present study aimed to assess the short and medium-term effects of Cu contamination on the soil fungal community. Two contrasting agricultural soils, an acidic sandy loam and an alkaline silt loam, were used for an eco-toxicological greenhouse pot experiment. The soils were spiked with a Cu-based fungicide in seven concentrations (0–5000 mg Cu kg^−1^ soil) and alfalfa was grown in the pots for 3 months. Sampling was conducted at the beginning and at the end of the study period to test Cu toxicity effects on total microbial biomass, basal respiration and enzyme activities. Fungal abundance was analysed by ergosterol at both samplings, and for the second sampling, fungal community structure was evaluated via ITS amplicon sequences. Soil microbial biomass C as well as microbial respiration rate decreased with increasing Cu concentrations, with EC_50_ ranging from 76 to 187 mg EDTA-extractable Cu kg^−1^ soil. Oxidative enzymes showed a trend of increasing activity at the first sampling, but a decline in peroxidase activity was observed for the second sampling. We found remarkable Cu-induced changes in fungal community abundance (EC_50_ ranging from 9.2 to 94 mg EDTA-extractable Cu kg^−1^ soil) and composition, but not in diversity. A large number of diverse fungi were able to thrive under elevated Cu concentrations, though within the order of *Hypocreales* several species declined. A remarkable Cu-induced change in the community composition was found, which depended on the soil properties and, hence, on Cu availability.

## Introduction

The infestation with foliar diseases such as downy mildew (*Plasmopara viticola*) and anthracnose (*Elisinoe ampelina*) can cause severe losses in grapevine (*Vitis vinifera* L.) production (Merrington et al. 2002). Copper (Cu)-based fungicides such as the Bordeaux mixture have been used to control these plant diseases since the end of the 19th century, and Cu compounds including Cu-oxychloride and Cu-hydroxide are still applied today in both, conventional and organic viticulture as well as in horticulture (Brun et al. 1998). As a consequence of the regular and frequent long-term foliar application of Cu-based fungicides, Cu has accumulated in many agricultural and viticultural soils. Especially in surface horizons through direct application, drift, or dripping of excess sprays from leaf surfaces (Chaignon et al. 2003) and because of its limited mobility, which is related to soil clay content and organic matter (Kabala and Singh 2001). High total Cu concentrations have been found in vineyards in France (up to 1,500 mg Cu per kg of dry soil), Australia (320 mg Cu kg^−1^), India (131 mg Cu kg^−1^), Italy and other countries (320 mg Cu kg^−1^) (Deluisa et al. 1996; Pietrzak and McPhail 2004). In typical Austrian viticultural regions, highest Cu concentrations have been reported for the Weinviertel and Wagram, with up to 831 mg Cu kg^−1^ and 888 mg Cu kg^−1^, respectively (Berger et al. 2012).

Plant protection against fungal diseases not only harms the target pathogens, but can potentially cause detrimental effects on a range of beneficial soil organisms, such as saprotrophic microbes (Peèiulytë 2001), and may lower terrestrial biodiversity. As a result, soil-borne plant pathogens might benefit from an impaired soil microbial community due to unspecific and non-target effects of fungicides.

Among the soil organisms, fungi and bacteria are generally the most studied. Although bacteria are predominant in quantity, fungal mass in the soil is of similar magnitude owing to their cell size (Blume et al. 2009). So far, the majority of metal contamination studies has focused on soil bacterial communities. Soil fungi, however, play a major role in the decomposition of complex plant or animal-derived compounds and thereby on soil biogeochemical cycling (Aguilar-Trigueros et al. 2015). Moreover, some soil-borne fungi show biocontrol activity against pests and diseases by antagonistic strategies (Pal and Gardener 2006). However, it is not entirely understood how Cu influences the fungal community structure and ecosystem functions in soil.

Copper is not only a toxic heavy metal but also an essential redox active transition metal, necessary for organism functions, e.g. the catalysis of oxidation by a number of enzymes (de Boer et al. 2012). As Cu^2+^, it is present in the cytochrome oxidase, the nitrite reductase of denitrifying bacteria and fungi as well as in oxygenases (Kandeler 2015). However, excessive Cu concentrations are potentially toxic through the catalytic formation of reactive oxygen species and the subsequent oxidative stress as well as by the oxidation of proteins, DNA, and lipids, and thereby causing cell death (Li et al. 2014) of organisms (MacKie et al. 2012).

As Cu pollution also exerts a potential risk to soil fertility and microbially driven soil ecosystem services such as nutrient cycling processes (Komárek et al. 2010; Wightwick et al. 2008), it is essential to evaluate Cu toxicity to soil biota. A dilemma in this context is that there are little alternatives for Cu fungicides in organic viticulture, while these systems heavily rely on beneficial microbes that develop and interact at soil–plant interfaces. It has been shown that Cu contamination can exert a strong effect on the soil microbial community, causing a decline of microbial biomass carbon (Li et al. 2015), basal respiration (Romero-Freire et al. 2016) as well as changes in metabolic quotient (Merrington et al. 2002), microbial activities and shifts in the microbial community structure (Li et al. 2014; Wang et al. 2007).

On the other hand, microbes have evolved resistance mechanisms in response to heavy metal stress (Vig et al. 2003; Zapotoczny et al. 2007), e.g. some fungi are resistant to Cu and have the ability to grow at concentrations that are toxic to other organisms (Gadd and Griffiths 1980; Peèiulytë 2001). This might be attributed to the fact that they are able to mobilize, sequester, or transform various ions (Gadd 2013; Gadd and Sayer 2000) thereby affecting the metals’ biogeochemical mobility.

As non-target effects of Cu-based fungicides the soil microbial processes and the soil fungal community are still not well understood, the present study aimed at quantifying the responses of selected microbial parameters with emphasis on the soil fungal community structure. A main objective was to identify fungal groups that show resistance and those that suffer from non-target effects.

## Materials and methods

### Experimental setup

Two contrasting agricultural soils both used for organic farming and exhibiting low background Cu concentrations, were sampled in spring 2015. The upper 20 cm were taken from the soils located in Lasberg (soil L, Upper Austria) and Deutsch Jahrndorf (soil D, Burgenland), which mainly varied in soil texture and pH (Table 1). A greenhouse pot experiment was set up using field moist, well homogenized soil. The pots (Ø 21 cm, height 20 cm) were filled with 4 L of soil and compacted to a soil bulk density of 1.2 g cm^−3^. The pots were rinsed twice (one week in between) with an amount of water equivalent to 200 % of the water holding capacity (WHC) to flush nitrogen that may have mineralized through the disturbance during the experimental set up. Two weeks later, six different concentrations (0, 50, 100, 200, 500, 1500, 5000 mg Cu kg^−1^) of a commercially available fungicide based on Cu(OH)_2_ (53.7% according to the product label; with a set of formulation agents that are not aimed to target activity and microbial community according to the manufacturer’s information) were applied as a suspension in water to both soils in five replicate pots each. The purity of the commercial fungicide was evaluated by measuring the Cu concentration and a set of other elements (Mn, Fe, Ni, Zn, Sr, Pb) after acid digestion of the fungicide powder. An open digestion of 0.2 g fungicide was performed with 65 % nitric- and 70 % perchloric acid (6:1, v/v, suprapur, Merck) according to ÖNORM L 1085 (2009), filtered (Whatman. 589/2, white ribbon) and analyzed by an inductively coupled plasma - mass spectrometer (ICP-MS, ELAN DRL-e SIEX, Perkin Elmer, Waltham, MA, US). The data are given in Supplementary Table S1. In addition, basic characterization of the fungicide was conducted, the pH was measured as outlined below. The electrical conductivity, organic carbon and total nitrogen were evaluated with standard methods as described elsewhere (Liu et al. 2017). The data are given in Supplementary Table S2. X-ray diffraction was used to obtain more information on the composition of the applied fungicide. The results are shown in Supplementary Fig. S1.

**Table 1:** Basic characteristics of the two studied soils

	Location	Province	Texture^a^	pH (CaCl_2_)	Sand	Silt	Clay	C_org_	N_total_	C:N	eCEC	Cu^b^	P^c^	K^c^
					%	%	%	%	%		cmol_c_ kg^−1^	mg kg^−1^	mg kg^−1^	mg kg^−1^
Soil D	Deutsch Jahrndorf	Burgenland	silt loam	7.5	29	55	17	1.76	0.18	9.8	22.03	8	122	402
Soil L	Lasberg	Upper Austria	sandy loam	5	66	26	8	1.64	0.17	9.9	5.91	2.3	87	129

^a^ FAO classification

^b^ in EDTA - Ethylenediaminetetraacetic acid

^c^ in CAL - calcium acetate lactate

For the sake of simplicity, we will further refer to the soils and treatments by indicating site and Cu amount applied as D0-D5000 and L0-L5000.

Each pot received ten seeds of the legume alfalfa (*Medicago sativa* L. cultivar. Plato) and no further fertilisation. Alfalfa (*Medicago sativa* L. cultivar. Plato) was chosen as a test plant, as legumes are essential cover crops in organic viticulture to improve soil N levels. For passive watering of the pots glass fibre wicks connected the soil in the pots with a 5-L bucket beneath containing artificial rain water (3 mg L^−1^ Ca; 50 % CaCl_2_ and 50 % CaSO_4_).

### Pot sampling

Each replicate of the greenhouse pots was sampled on May 21st 2015 (28 days after Cu application) and a second time during plant growth on August 7th (106 days after Cu appplication). Bulk soil samples were taken with a 1 cm stainless steel auger over the whole pot height and were homogenized by sieving (<2 mm) to remove most of the fine roots and stored at 4 or −20 °C before analyses.

### Laboratory analyses

#### Soil properties

Soil Cu was extracted with EDTA and CaCl_2_, respectively. Five g dry soil was extracted with 50 mM EDTA in a 1:10 ratio shaking for 2 h followed by filtration (Schleicher & Schuell, Dassel, Germany) according to the Austrian Standard OENorm-L1089 (2005). 10 mM CaCl_2_ extracts were prepared from 2.5 g air dried soil, which equilibrated in 50 ml solution overnight before shaking for 3 h, filtration (Munktell, 14/N) (Houba et al. 2000). In both extracts, Cu was measured with flame atomic absorption spectroscopy (AAS, AAnalyst 400, Perkin Elmer, MA, US).

The pH value was measured with 2 g air-dried soil in 25 mL of a 0.01 M CaCl_2_ solution (OENorm-L1083 2006). Cation exchange capacity (CEC) was extracted with 0.1 M BaCl_2_ at a 1:20 w/v ratio of a according to OENorm-L1086 (2001), and the exchangeable cations (Ca, Mg, K, Na, Al, Fe, Mn) were determined via inductively coupled plasma optical emission spectrometry (ICP-OES). Plant available potassium (K_CAL_) and phosphorus (P_CAL_) were extracted with calcium acetate lactate (CAL) according to OENorm-L1087 (2004). K_CAL_ was analysed by AAS (Perkin Elmer 2100), and P_CAL_ was measured photometrically based on the phosphomolybdate blue reaction (Schinner et al. 1996).

#### Microbial biomass

Chloroform fumigation of soil for 24 h kills microbial cells with the release of cytoplasm (Alessi et al. 2011). The soil samples were treated with the chloroform fumigation extraction (CFE) method as described by Schinner et al. (1996) to determine dissolved organic carbon (DOC), microbial biomass carbon (SMBC) and nitrogen (SMBN). Fumigated and non-fumigated samples were measured with an automated TOC/TN analyser (TOC-V CPHE200V, linked with a TN-unit TNM^−1^ 220 V, Shimadzu Corporation, Kyoto, Japan) according to Brandstätter et al. (2013).

#### Ergosterol

Two g of freeze dried soil sample was used for extraction of lipids according to Frostegård (1993). Lipids were extracted and fractionated by solid phase extraction using Silica gel columns (Isolute® SI 500 mg of 3 mL, Biotage, Sweden), and the neutral lipids were recovered to measure ergosterol. The neutral lipids were eluted in 1 M methanol, and transferred into brown vials for measurement with high performance liquid chromatography (HPLC, 1290 Infinity, Agilent Technologies Inc., Santa Clara, CA, US) coupled with a UV detector (λ = 282 nm). The C18 column was an Eclipse (Agilent Technologies Inc.) with a size of 2.1 × 50 mm.

#### Basal respiration

For basal respiration measurement, 2 g of moist soil was weighed into headspace vials and moistened with distilled water to 40 % WHC. The CO_2_ in the headspace was measured with a gas chromatograph (GC, Agilent 7890 A) connected with a headspace sampler (Agilent 7697 A) and a flame ionization detector (FID Ni-cat) at an oven temperature of 300 °C with the following settings: 35 mL min^−1^ He and 350 mL min^−1^ synthetic air, 11.9 mL min^−1^ makeup flow, carrier gas He. An amount of 200 µL gas was injected and separated on a GS-carbonplot column (30 m widebore, inner diameter 0.32 mm, film 3 µm, JW, Santa Barbara, CA, USA). Extraction conditions of the headspace sampler: 3 min at 25 °C. Calibration was conducted with externally certified gas standards (CO_2_: 251 ppm, 501 ppm, 990 ppm). Samples were analysed twice, before and after incubation (8 h at 22°C) and CO_2_ concentration was calculated with Chemstation 32 quantitative mode. Basal respiration rate was calculated according to Creamer et al. (2014).

#### Potential oxidative enzyme activity

The activity of phenoloxidase and peroxidase was measured photometrically in microplates based on standard methods (Sinsabaugh et al. 1999), using L-3,4-dihydroxyphenylalanin (L-DOPA, Sigma-Aldrich, Vienna, Austria). One g of soil was suspended in 100 mL 100 mM sodium acetate buffer (pH 5.5) and homogenized with an ultrasonic probe in continuous mode at 10 % energy for 1 min (HD2200 with 200 W, Bandelin electronics, Berlin, Germany). An aliquot of 900 µL was mixed with a 20 mM L-DOPA solution in a 1:1 ratio, by shaking for 10 min on a horizontal shaker. Then the mixtures were centrifuged and 250 µL of the supernatant pipetted into microplates in 3 replicates. For peroxidase measurement, wells additionally received 10 μL of a 0.3% H_2_O_2_ solution (see also Keiblinger et al. 2012). Absorption was read at the beginning and after incubation at a wavelength of 450 nm, and potential phenoloxidase and peroxidase activities calculated according to German et al. (2011).

#### DNA extraction

For DNA extraction, 0.5 g of sieved soil was added to 1.5 mL of LifeGuard Soil Preservation Solution (MO BIO, Carlsbad, CA, US) and stored at 4 °C until further processing. Half of the suspension was added to the wells of a PowerSoil-htp 96 Well Soil DNA Isolation Kit (MO BIO). After centrifugation and removal of the supernatant, the centrifugation protocol was followed according to the manufacturer’s instructions. The DNA was quantified with the Quant-iT^TM^ dsDNA Assay Kit (ThermoFisher Scientific, Waltham, MA, US).

#### Library preparation and Illumina MiSeq sequencing

For profiling of the fungal communities, the ITS2 region was amplified with primer pair ITS3 and ITS4 as recommended by Tedersoo et al. (2015). For both primers, equimolar mixes were used to increase coverage of the fungal kingdom: the ITS3Mix containing five different primers (Tedersoo et al. 2014) and the ITS4Mix containing the original ITS4 (White et al. 1990) and a degenerate version thereof (for primer details see Supplementary Material Table S2). All primers contained the universal 5’ tails as specified in the Nextera library protocol from Illumina. The PCR reactions in a 20 µL mixture contained 4 µL 5 x Phusion HF, 1 µL of each primer (10 µM), 0.4 µL dNTP mix (10 mM), 0.2 µL Phusion Polymerase, 1 µL of tenfold diluted DNA and 12.4 µL PCR-grade water. Thermal-cycling conditions were as follows: an initial denaturation of 3 min at 95 °C, 35 cycles of 30 s at 95 °C, 30 s at 55 °C and 30 s at 72 °C with a final elongation of 72 °C for 5 min. All reactions were carried out in duplicate and pooled after amplification. The AMPure XP beads (Beckman Coulter, Brea, CA, US) were used to purify the ITS2 amplicon away from free primers and primer dimer species. Afterwards the Nextera XT Index Kit (Illumina, San Diego, CA, US) was used to attach dual indices and Illumina sequencing adapters. The Index PCR reaction in a 50 µL mixture contained 0.5 µL Phusion, 5 µL of each primer (10 µM), 10 µL HF Buffer, 1 µL dNTP mix (10 mM), 5 µL of DNA and 23.5 µL PCR-grade water. Thermal-cycling conditions were as follows: an initial denaturation of 3 min at 95 °C, 8 cycles of 30 s at 95 °C, 30 s at 55 °C and 30 s at 72 °C with a final elongation of 72 °C for 5 min. Again AMPure XP beads were used to clean up the final library before quantification by Quant-iT dsDNA Assay Kit, high sensitivity (ThermoFisher Scientific). Before being sequenced on the Miseq platform (Illumina), all samples were pooled in equimolar quantities. Samples were sent for sequencing to the sequencing core facility at the Vienna Biocenter (VBCF-NGS, Vienna, Austria).

### Data evaluation and statistical analysis

Sequence data files were received from the sequencing core facility as two fastq files for each sample from the forward and reverse reads based on the given barcodes. Only forward reads were considered in the downstream analysis. USEARCH program suite (Edgar 2010) was used for filtering short sequences with minimal length of 280 bp. FASTX toolkit script fastx_barcode_splitter.pl was used to sort out project specific fungal sequences and USEARCH scripts were used for chimera detection, filtering underrepresented sequences ( < 10), clustering and counting sequences per cluster given a 97% sequence similarity. The results were a sequence file of Operational Taxonomic Units (OTU) holding one representative sequence per cluster and a table with counts of each OTU per sample. The OTU sequences were aligned using Clustal Omega and a PhyML tree (Guindon and Gascuel 2003) was calculated and imported into R (R-Development-Core-Team 2008). Taxonomic affiliation of OTUs was done with the UTAX script against the UNITE database (Kõljalg et al. 2013), while manual editing of the data increased phylogenetic information. Non-fungal sequences and samples containing less than 3500 fungal sequences were removed from further analyses.

Shannon indexes were calculated using R-package ‘vegan’ and summarized by Cu treatment for each soil. For Principal Coordinate Analysis, the distances between fungal communities in respect to copper treatment in each soil were calculated as generalized UniFrac distances using R package ‘GUniFrac’ (Chen et al. 2012) with an alpha value of 0.5 to avoid domination by overabundant species. Classical multidimensional scaling of the resulting data matrix was performed to obtain a 2-dimensional representation of these distances (Gower 1966). High throughput sequencing files have been deposited at NCBI SRA under the accession number: SRP092758

Dose response relationships were evaluated for the data with a standard Hill model (by a four parametric logistic (4PL) curve using Sigma Plot 12.0 with the equation (Eq. 1) to calculate the half maximum effective concentration (EC_50_) (Dawson et al. 2012).

1 $$y = min + \frac{{(max - min)}}{{1 + \left( {\frac{x}{{EC50}}} \right)^{ - Hillslope}}}$$

Where y is the dependent variable or microbial parameter that is affected by Cu, x is the independent variable, the different doses/concentrations of Cu. Minimum asymptote is indicated as “min” (response at no added Cu), whereas maximum asymptote is indicated by “max”(response value at infinite Cu doses). EC_50_ is the inflection point, where the half effect dose is reached for the soil microbial parameter of interest. The Hillslope refers to the steepness of the curve.

## Results

### Variations in physico-chemical and microbial soil characteristics across treatments

The two soils used in our experiment were similar in organic C, total N and C:N ratio (Table 1). Soil D had higher pH, effective cation exchange capacity, calcium-acetate-lactate-extractable K and P, and finer texture compared to soil L. Four and fifteen weeks after addition of Cu in form of Funguran, EDTA-extractable Cu was similar in the two soils (Table 2); however, CaCl_2_-Cu was different in the two soils with higher concentrations for soil L, as indicated by higher CaCl_2_-Cu / EDTA-Cu ratios in Table 2.

**Table 2:** EDTA-extractable Cu and the ratio of CaCl_2_-Cu to EDTA-Cu of the two investigated soils for both samplings

		1st sampling		2nd sampling	
soil	Cu spiked	EDTA-Cu	CaCl_2_–Cu / EDTA-Cu	EDTA-Cu	CaCl_2_–Cu / EDTA-Cu
	mg kg^−1^	mg kg^−1^	10^−3^	mg kg^−1^	10^−3^
L	0	1.86 ± 0.10	45.7	2.84 ± 0.22	64.5
	50	39.55 ± 12.83	10.3	29.89 ± 5.46	5.10
	100	81.98 ± 10.46	12.7	57.06 ± 3.63	2.67
	200	215.5 ± 33.54	14.4	100.4 ± 7.76	4.17
	500	605.8 ± 117.4	56.0	274.4 ± 37.91	17.9
	1500	2229 ± 413.0	24.3	973.2 ± 104.7	18.0
	5000	7082 ± 1043	10.4	3437 ± 92.13	15.1
D	0	7.516 ± 0.09	23.7	13.33 ± 1.60	7.71
	50	48.35 ± 10.76	0.835	54.14 ± 3.08	1.01
	100	77.48 ± 7.97	0.569	91.00 ± 7.82	0.599
	200	171.36 ± 37.71	0.503	132.4 ± 19.71	0.517
	500	632.0 ± 34.46	0.491	397.2 ± 42.85	0.553
	1500	1663 ± 216.4	0.439	1752 ± 122. 9	0.312
	5000	2742 ± 531.9	0.505	3315 ± 206.4	0.371

The table shows the means, standard errors (SE) with n = 5

**Fig. 1:**
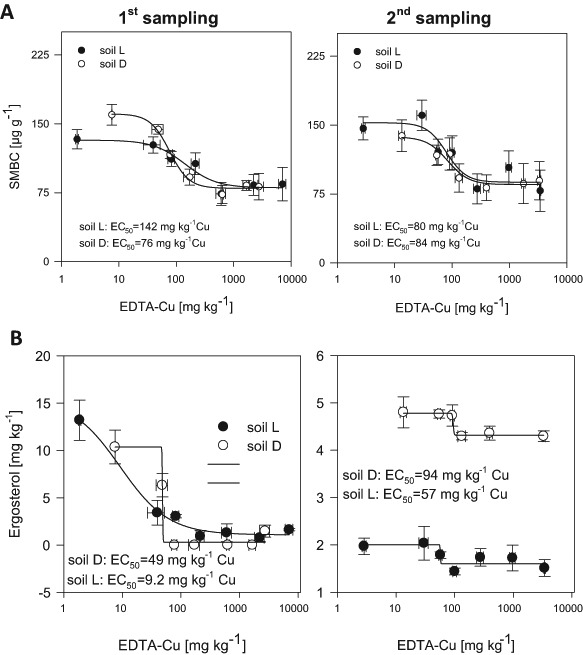
**a** Soil microbial biomass carbon (SMBC) **b** Ergosterol in the two different soils at both sampling times (1st sampling and 2nd sampling, 4 and 15 weeks after Cu addition, respectively) in dependence of EDTA-extractable Cu. Error bars indicate standard error; n = 5

SMBC was sensitive to increasing Cu concentrations at both sampling times and for both soils. A clear dose response relationship for Cu and SMBC was seen at lower Cu concentrations at both sampling times (see Fig. 1a, Supplementary Table S4). The half maximal effective concentration (EC_50_), for the first sampling was 76 mg kg^−1^ EDTA-Cu and 142 mg kg^−1^ EDTA-Cu for soil D and soil L respectively (Fig. 1a, Supplementary Table S4). For the second sampling the EC_50_ values slightly changed to around 80 mg kg^−1^ EDTA-Cu for both soils (Fig. 1a, Supplementary Table S4); these results are consistent with a negative correlation of SMBC with EDTA-Cu over both soils (see Supplementary Table S2). The response levelled off at an application rate of 500 mg Cu kg^−1^ soil (see Fig. 1a**)**.

The ergosterol concentration followed a similar trend as SMBC for the first sampling (Fig. 1b) albeit with lower EC_50_ values than SMBC, i.e. 49 and 9 mg kg^−1^ EDTA-Cu for soil D and soil L, respectively (Fig. 1b, Supplementary Table S4). At the first sampling, ergosterol started to decline at the lowest application rate and seemed to be especially sensitive to Cu; for soil D, the values were almost approaching the limit of detection. At the second sampling, the EC_50_ values increased for both soils by approximately 50 mg kg^−1^ Cu (Fig. 1b). Interestingly, the maximum values declined and minimum values increased compared to the first sampling (Supplementary Table S4). At the second sampling higher ergosterol concentrations and a higher EC_50_ value were observed for soil D compared to soil L.

### Short to medium-term effects of copper application on respiration and enzyme activities

**Fig. 2:**
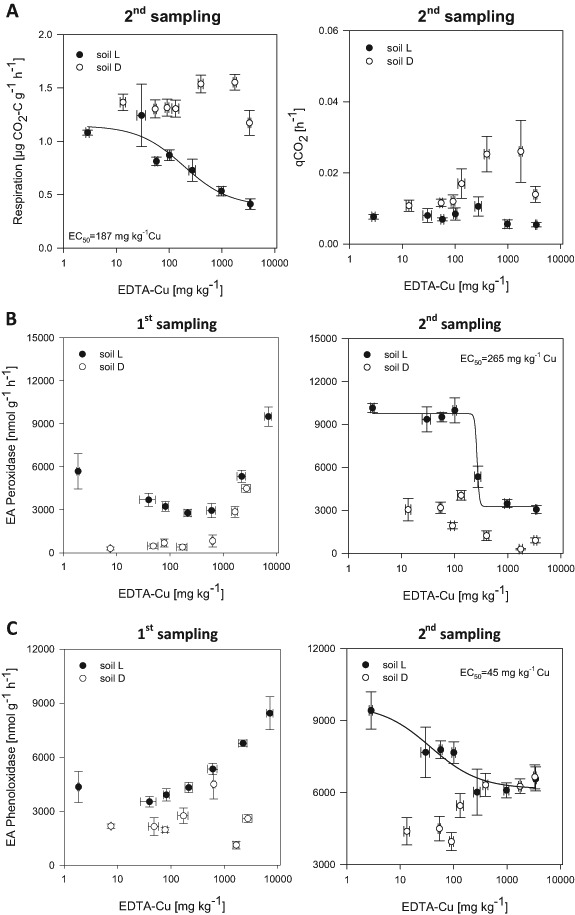
**a** Basal respiration rate and metabolic quotient (qCO_2_) in the two different soils at the second sampling 15 weeks after Cu addition) **b** Enzyme activity (EA) of peroxidase and **c** phenoloxidase in the two different soils at both sampling times (1st sampling and 2nd sampling, 4 and 15 weeks after Cu addition, respectively) in dependence of EDTA-extractable Cu. Error bars indicate standard error; n = 5

Basal respiration rate was only measured at the second sampling. Consistent with SMBC, metabolic activity in terms of basal respiration decreased with increasing Cu, especially in soil L (Fig. 2a). For soil L, a clear dose response relationship was observed, and the EC_50_ value for EDTA-Cu was determined at 187 mg kg^−1^ (Fig. 2a, Supplementary Table S5); correspondingly, a significant negative correlation of EDTA-Cu and respiration was observed for soil L (Supplementary Table S6). For soil D, the values at lower concentrations remained almost constant at 1.3 µg CO_2_-C g^−1^ h^−1^ and even rose at 500 and 1500 mg Cu kg^−1^ addition (Fig. 2a). Basal respiration in soil D had a similar trend as the shoot biomass (Supplementary Fig. S2). For soil L and over both soils, basal respiration was significantly positively related to shoot and root biomass (Supplementary Table S3). The respiration rate related to SMBC, i.e. the metabolic quotient qCO_2_, showed no response to Cu in soil L (Fig. 2a) indicating that basal respiration and SMBC were similarly reflecting the active microbial community. In soil D, the increased respiration at 500 and 1500 mg Cu kg^−1^ resulted in a trend of increasing qCO_2_.

Both oxidative enzyme activities showed complex responses to Cu addition (Fig. 2b & c). In soil L, phenoloxidase increased significantly for the first sampling, while for the second sampling a decrease up to an application rate of 500 mg Cu kg^−1^ was observed (Fig. 2) and an EC_50_ value of 45 mg kg^−1^ EDTA-Cu was calculated (Supplementary Table S5). In soil D, phenoloxidase showed the highest activity at 500 mg Cu kg^−1^ for the first sampling and an increasing trend with Cu application for the second sampling.

For peroxidase at the first sampling, there was a decline up to 500 mg Cu kg^−1^, but rising activities at higher Cu concentrations in soil L, while the activities remained similar up to 500 mg Cu kg^−1^ and then also increased in soil D (Fig. 2c). For the second sampling, the opposite trend was observed for both soils, i.e. high peroxidase activities up to 200 mg Cu kg^−1^ and a significant decline with higher Cu concentrations. For soil L an EC_50_ value of 265 mg kg^−1^ EDTA-Cu was calculated (Fig. 2c, Supplementary Table S5).

### Profiling of the fungal community

The Illumina MiSeq sequencing approach resulted in sufficient fungal sequences for all but one sample from soil L. A high number of low efficiency amplification in soil D, however, led to the exclusion of many samples from further analyses due to low numbers of sequences.

Soil L tended to have higher numbers of fungal OTU per sample (60-116 OTUs per sample) compared to soil D (38-77). Similarly, higher numbers of fungal OTUs were found after pooling of data from replicates in soil L (180-140 OTUs per treatment) compared to soil D (80-88), but lower numbers of OTUs per treatment in soil D might have been influenced by lower numbers of community profiling data from soil D due to missing data (Table 3). No significant differences in OTU richness were found between treatments in any soil.

**Table 3:** Richness and diversity of soil fungal communities

soil	Cu spiked	n	OTU/sample	OTU/treatment	most abundant OTU	H'
	mg kg^−1^				%	
D	0	2	62.5 ± 0.4	81	71.3	1.5 ± 0.42
	50	3	62.7 ± 2.5	88	82.6	1.1 ± 0. 39
	100	3	64.0 ± 4.2	88	85.8	1.0 ± 0.10
	200	4	61.0 ± 1.8	84	56.8	1.8 ± 0.19
	500	3	60.7 ± 6.3	81	73.0	1.3 ± 0.27
	1500	2	57.5 ± 13.8	85	43.1	1.7 ± 0.13
	5000	2	62.0 ± 2.8	80	29.5	1.7 ± 0.04
L	0	5	98.0 ± 2.5	137	43.9	2.4 ± 0.33
	50	4	106.3 ± 2.5	135	18.3	2.8 ± 0.09
	100	5	92.4 ± 3.0	136	23.4	2.5 ± 0.08
	200	5	84.4 ± 1.0	118	31.1	2.4 ± 0.02
	500	5	74.6 ± 0.2	120	56.3	1.7 ± 0.07
	1500	5	72.6 ± 3.0	123	37.0	1.2 ± 0.20
	5000	5	103.2 ± 3.3	140	24.1	2.4 ± 0.39

n: number of samples with sufficient reads for molecular analyses

OTU/sample: average number of OTU per sample ± standard error

OTU/treatment: number of OTUs per treatment after pooling of data from replicates

most abundant OTU: relative abundance in % of most abundant OTU in pooled data from replicates

H’: Shannon Index for fungal diversity in soil samples, average ± standard error from replicates

The fungal communities in soil D were less even than in soil L. The most abundant OTU in untreated soil D covered more than 70% of all reads. Maximum OTU abundance further increased to more than 80 % in the D50 and D100 treatments and dropped to below 30% in the D5000 treatment. Soil L was generally more even. Most abundant OTUs covered between 18.3 % (L50) and 56.3 % (L500) of all reads.

In soil D, Shannon diversity did not change with Cu application, and in soil L a significant reduction was only overserved for the two highest application rates (1500 and 5000 mg kg^−1^, Table 3).

**Fig. 3:**
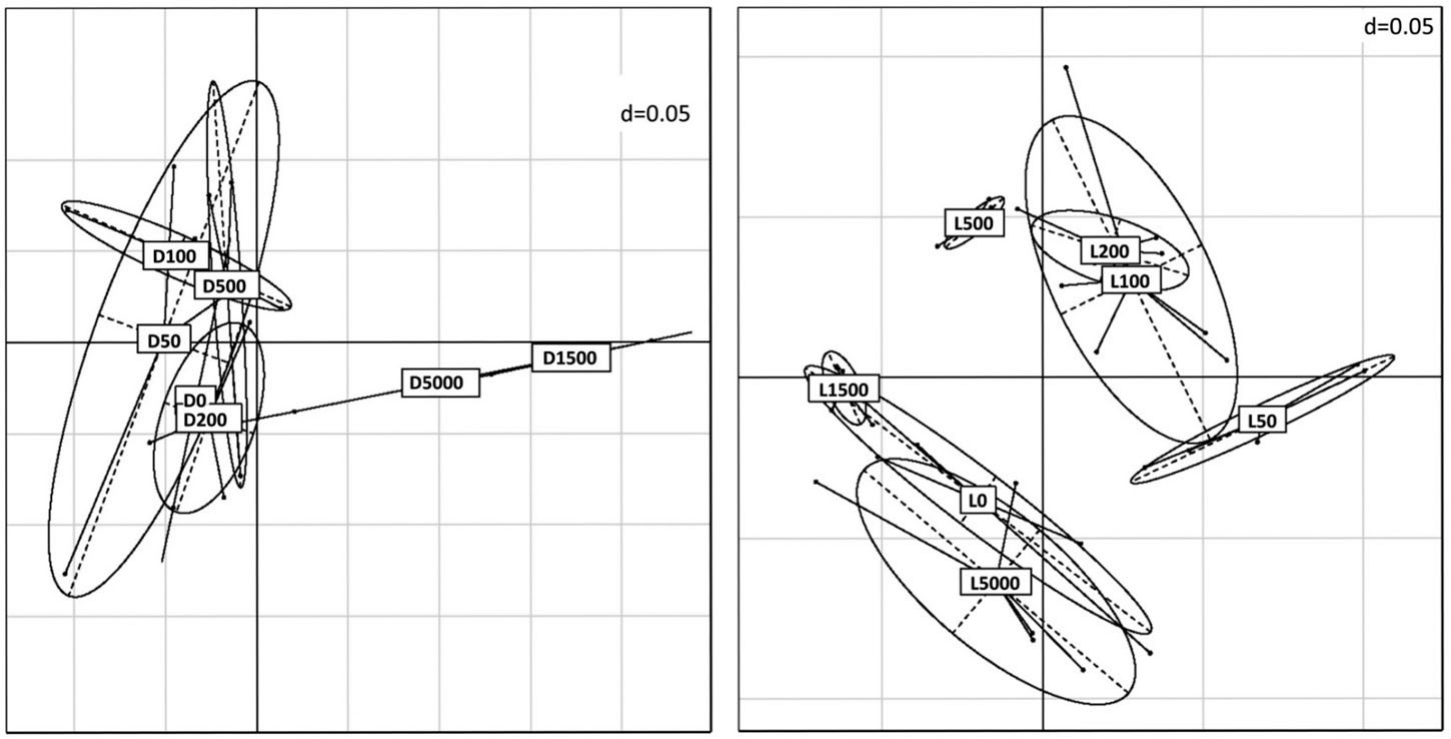
Principal coordinate analysis (PCoA) derived from generalized UniFrac distances with an alpha value of 0.05 based on the 97% OTU level of the fungal community compositions across different copper treatments in soil D (left) and soil L (right) at the second sampling. Ellipses: represent ellipses of dispersion, dotted lines: axes of ellipses, dots: community of sample, grid: distance = 0.05

In soil D, the fungal communities found in samples treated with high amounts of Cu (1500 and 5000 mg kg^−1^) deviated from the ones in the other treatments (Fig. 6), whereas in soil L, communities with medium Cu treatment (50–500 mg kg^−1^) were separated from no or high (1500 and 5000 mg kg^−1^) treatments (Fig. 3).

**Fig. 4:**
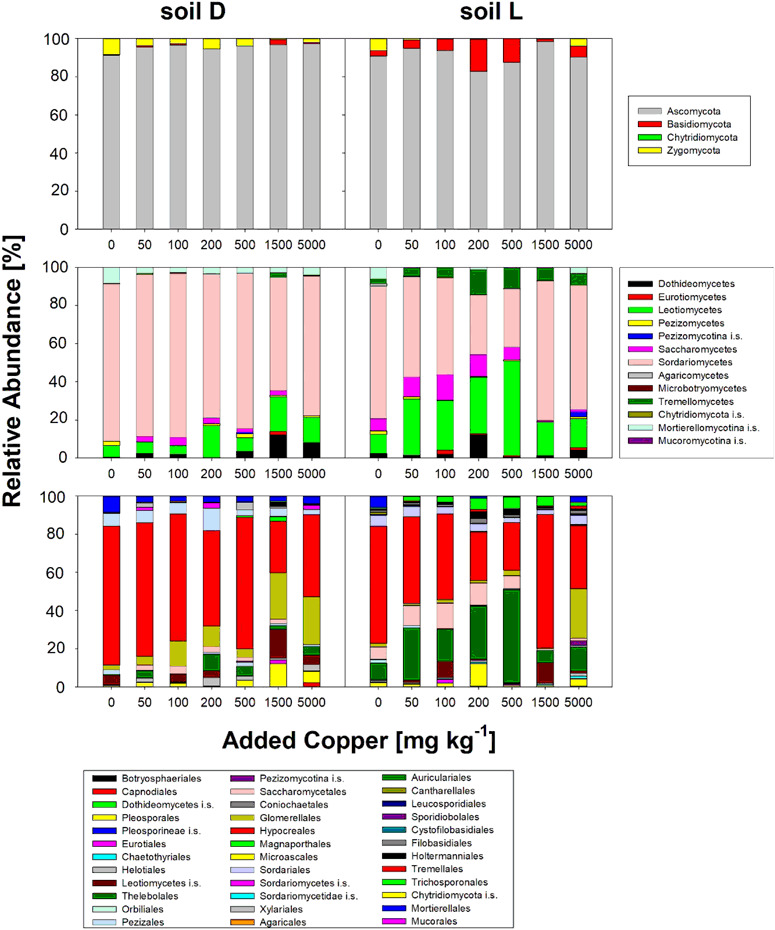
Relative abundance of fungal phyla (top panel) classes (middle panel) and order (bottom panel) with increasing concentrations of added Cu, for soil D (left) and soil L (right)

All communities from both soils and all treatments were dominated by Ascomycota with a relative abundance of more than 80% (Fig. 4). The most abundant classes were Sordariomycetes and Leotiomycetes with high abundance in soil L for treatments L500 and L1500 (Fig. 4). The two most abundant orders across all samples were Hypocreales (Fig. 4) and Glomerellales with OTU read abundances of ~50 and 9%, respectively.

**Fig. 5:**
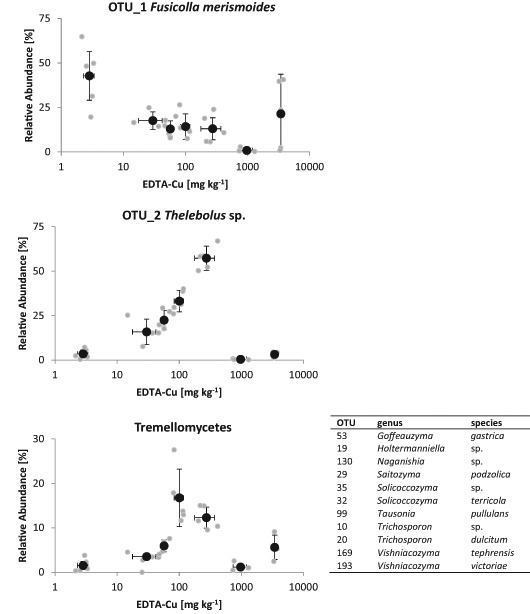
Relative abundances of OTU_1 (*Fusicolla merismoides*, top panel), OTU_2 (*Thelebolus* sp., middle panel) and the sum of OTUs classified as *Tremellomycetes* (detailed OTUs, including genus and species that account to this class see Table next to Figure) in soil L are shown for single samples (grey dots ) and averages from treatments (black dots • with standard deviations)

**Fig. 6:**
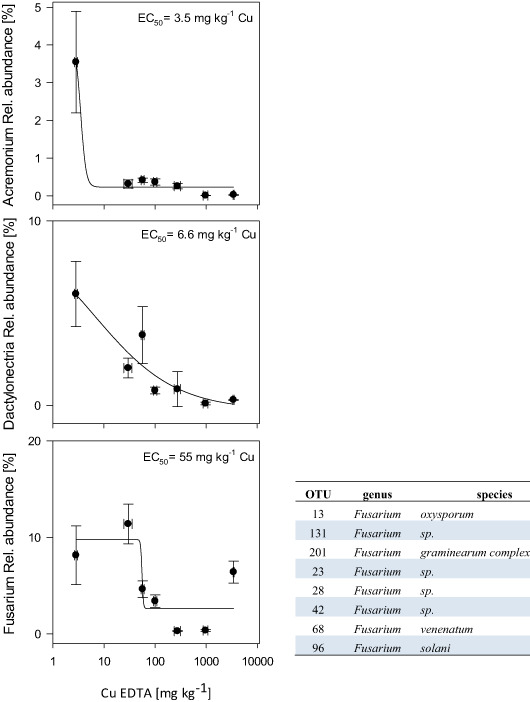
Relative abundances of Acremonium OTU_38 (*Acremonium Antarctica*, top panel), Dactylonectria OTU_21 (*Dactylonectria sp*., middle panel) and the sum of OTUs classified as *Fusarium* (detailed OTUs, including genus and species that account to this class see Table next to Figure) in soil L averages from treatments (black dots • with standard errors), as well as dose response relationships, and EC50 values

Soil L was dominated by OTU_1 – *Fusicolla merismoides* – in the absence of added Cu. With the addition of Cu, OTU_2 – *Thelebolus* sp. – became dominant, but was again replaced by OTU 15 – *Emericellopsis* sp. – in L1500 (Fig. 5) and OTU_1 and OTU_14 – *Plectosphaerella* sp. – in L5000. The hypocrealean, potentially root pathogenic genera *Dactylonectria* and *Ilyonectria* (OTU_7, OTU_9, OTU_21) were highly abundant in soil L but were nearly absent at the highest Cu concentration (Fig. 6). Furthermore, a group of basidiomycetous yeasts or dimorphic fungi in the class Tremellomycetes was highly abundant in soil L, especially at intermediate Cu concentrations (Figs. 4 and 5).

Soil D was strongly dominated by OTU_1 – *Fusicolla merismoides* – except in the D1500 and one D5000 sample. These samples were dominated by OTU_14 – *Plectosphaerella* sp. *Dactylonectria* and *Ilyonectria* (OTU_7, OTU_9 and OTU_21), which were abundant in soil L, were nearly absent in soil D.

In soil L, *Acremonium*, *Dactylonectria*, and *Fusarium* OTUs strongly declined with rising Cu concentrations (Fig. 6). The EC_50_ was very low for *Acremonium* and *Dactylonectria* with values of 3.5 and 6.6 mg kg^−1^ EDTA-Cu, respectively. For the sum of OTUs that belong to the genus of Fusarium, the EC_50_ concentration was 55 mg kg^−1^ EDTA-Cu (Fig. 6, Supplementary Table S7).

## Discussion

### Short to medium-term effects of Cu addition on soil microbial and fungal biomass

Soil microbial biomass carbon (SMBC) was reduced in both soils at both sampling times after Cu application. This is likely due toxic effects of excess Cu (Gessler et al. 2011; Mackie et al. 2012) and renders SMBC as a useful indicator for soil health, providing valuable information about the total community (Li et al. 2015; Shade et al. 2012). Even in the long term of 50 years, Cu based fungicide contamination in different soils (12–3000 mg Cu kg^−1^) has been found to impair fungi, bacteria and actinomycetes (Hussain et al. 2009). While the EC_50_ value of soil D stayed rather constant, soil L seemed to respond to Cu at even lower concentrations at the second sampling, which may be an effect of its lower pH value.

High sensitivity of the total fungal community was observed at the first sampling time, reflected by lower EC_50_ values for the ergosterol content compared to total SMBC (Fig. 1b, Supplementary Table S4). Differences between EC_50_ values for ergosterol and SMBC were less pronounced at the second sampling. Fungal biomass seems to adapt to the added Cu, as the EC_50_ values increased for both soils from the first to the second sampling (11 weeks). On the other hand, at higher application rates ergosterol concentration were higher at the second sampling than at the first sampling (especially for soil D), which indicates adaption of fungi to Cu (Fig. 1b, Supplementary Table S4). The latter has been reported by several ecotoxicological studies as a response to Cu addition (e.g. Li et al. 2014). The observed pattern suggests a slow adaptation of the fungal community to the toxic effects of Cu. Reduced nutrient competition by bacteria and provision of additional nutrients from dead bacteria might have contributed to the increase in fungal biomass after prolonged incubation, and several mechanisms of fungi to cope with high heavy metal concentrations have previously been identified (Gorfer et al. 2009; Vig et al. 2003; Zapotoczny et al. 2007). In soil D, which is characterized by a higher pH, soil organic matter and clay content, and consequently lower availability of Cu, higher ergosterol concentrations were observed at the second sampling compared to soil L. This also fits to the results of plant biomass, which was less affected by Cu in soil D (Supplementary Fig. S2).

The observed trends are in line with the finding that bacteria and fungi respond differently to Cu in soil, with effects strongly influenced by soil characteristics (Rajapaksha et al. 2004). For example, soil texture might influence the Cu effect on the microbial community, as applications of 200 and 2000 mg kg^−1^ CuSO_4_ reduced fungal populations in loamy soils, but stimulated fungi in sandy soils (Hemida et al. 1997). However, ecotoxicological studies using CuSO_4_ also need to consider soil pH, as high concentrations have been shown to acidify the soils with low buffering capacity (Brandt et al. 2010; Fernández-Calviño and Bååth 2016; Rajapaksha et al. 2004), generally favoring fungi (Rousk et al. 2009). Thus, higher cumulative fungal growth in CuSO_4_ contaminated soil as described earlier are not fully unexpected (Fernández-Calviño and Bååth 2016; Rajapaksha et al. 2004). Besides, a stronger effect by pH than by Cu on the bacterial community has been reported for a study with a factorial design (de Boer et al. 2012). In the present study, Cu was applied in form of Cu(OH)_2_ and resulted in a constant soil pH up to 500 mg kg^−1^ Cu applied, but a significant increase in the highest application rates (up to 5.9 and 7.7 in soils L and D, respectively). While the pH shifts in the current experiment would not favor fungal development, soil pH also strongly determines the metal availability and therefore toxicity to the soil microbial community.

### Short to medium-term effects of Cu addition on respiration and enzyme activities

Basal respiration, which was only measured at the second sampling, followed a similar trend as SMBC, resulting in a stable metabolic quotient throughout the treatments for soil L. The decline in respiration gives an EC_50_ value of 187 mg kg^−1^ EDTA-Cu for soil L. This is similar to other short-term respiration results reported in response to Cu addition (e.g. Fernández-Calviño and Bååth 2016), and may be associated with heavy metal-induced microbial die off or complex formation with their substrates, which are then less available for energy production (Landi et al. 2000). The metabolic quotient has been described as an indicator for the levels of stress through contamination (Khan and Scullion 2002). From this, one might conclude that the microbial activity was more stressed in response to Cu in soil D than in soil L, where higher respiration and qCO_2_ were observed. However, this is controversial to the characteristics of soil D (Table 1) having higher pH, soil organic matter and clay content, which cause lower availability of Cu due to sorption or formation of complexes (Giller et al. 1998). In addition, also plant biomass was less affected by Cu in soil D (Supplementary Fig. S2). However, root exudates still available at higher Cu concentrations can provide substrate for microbial metabolism. This C substrate is most probably rather allocated for maintenance than for microbial growth, consequently, lowering substrate utilization efficiency and increasing qCO_2_ (Bahemmat et al. 2015; Liao and Xie 2007).

Oxidative enzymes showed an increase in activity at the first sampling, 4 weeks after Cu application. Indeed, phenol- and peroxidase can be deployed by bacteria and fungi for the mitigation of toxicity effects of Cu (Sinsabaugh 2010). When Cu becomes toxic, reactive oxygen radicals are favored (Zapotoczny et al. 2007), which may enhance the activity of oxidative enzymes. At the second sampling, 15 weeks after Cu application, peroxidase activity started to decline at a threshold concentration of 500 mg Cu kg^−1^ in both soils (Fig. 2b). This decline is probably due to a toxicity effect on alfalfa (Supplementary Fig. S1). Although the secretion of oxidoreductases such as lignin peroxidase and manganese peroxidase has been observed mainly in white rot fungi (Kuramae et al. 2013), peroxidase can also be excreted by bacteria, Actinobacteria and plant roots to stimulate degradative processes. In fact, root exudates of alfalfa, have been identified as an important source of oxidoreductases with lower activities as a consequence of lower root biomass (Gramss and Rudeschko 1998). The latter is also supported by our results. Even though bulk soil was sampled, the effect of plants in the pots can be observed at the second sampling compared to the first, where in both soils a strong reduction of oxidative enzymes was observed, along with a strong decline in plant biomass due to Cu application (Supplementary Fig. S1).

### Short to medium-term effects of Cu addition on the soil fungal community

The soil fungal community was highly uneven across both soils and all treatments, which has previously been described as a general feature of fungal communities (Fierer et al. 2007), but such a strong dominance of single taxa has not been found before in soil. Consequently, fungal diversity was lower than found in other studies for agricultural soils (e.g. Klaubauf et al. 2010). In our experiment soil homogenization before potting and planting might have contributed to the dominance of a few well adapted species. Also, pot experiments are expected to be less stratified vertically and horizontally. A similar tendency to high abundances of single taxa has been found in decomposing leaves in streams (Duarte et al. 2015). Fungal diversity as estimated by Shannon’s Index*’* was only slightly reduced in soil L upon addition of 500 or 1000 mg kg^−1^ Cu, but remained largely unaffected in the other treatments and in soil D. Little influence on fungal diversity, but changes in community structure by heavy metal contamination were also found in previous studies, where long-term effects in forest soils were studied (Hui et al. 2011; Op De Beeck et al. 2015).

All communities from both soils and all treatments were dominated by Ascomycota (Fig. 4), which is common in agricultural soils (Domsch and Gams 1970; Klaubauf et al. 2010; Moll et al. 2016). Stronger effects of Cu addition on fungal communities were seen in soil L, which can be explained by the higher bioavailability of Cu as indicated by the higher ratios of CaCl_2_-Cu /EDTA-Cu compared to soil D (Table 2). The most abundant class were Sordariomycetes with decline in relative abundance from L0 to L500; this was accompanied by the development of Leotiomycetes in soil L (Fig. 4). Both of these classes have been mentioned to be cellulase producing (Schneider et al. 2012), underlining their potential importance in soil C-cycling.

The most abundant species in the whole dataset, *Fusicolla merismoides* (formerly *Fusarium merismoides*, see also Figs. 5 and 6), is considered a common, saprotrophic soil fungus, which is also found in polluted water (Gräfenhan et al. 2011). It dominated the fungal community in soil L in the absence of Cu and in soil D in most treatments. The observed pattern suggests an r-strategy for soil colonization by *F. merismoides*, which might have been promoted by soil homogenization (see above) and which is especially successful in the absence of more competitive colonizers.

In soil L, *Thelebolus* sp. became more dominant at intermediate Cu levels (Fig. 5) which partially explains the separation of samples L50, L100, L200 and L500 from samples with low – L0 – and very high – L1500 and L5000 – Cu-concentrations in PCoA (Fig. 3). This pattern indicates a certain level of Cu resistance, which provides a competitive advantage at intermediate Cu levels, but does not allow good growth at high Cu concentrations. *Thelebolus* is described as a coprophilus genus with a good adaptation to lower temperatures (Wicklow and Malloch 1971). It seems to show a preference for agricultural sites as it was also found in a managed meadow and a pasture, but not in a cork oak forest and vineyards in Sardinia (Orgiazzi et al. 2012). Both agricultural sites, the managed meadow and the pasture, mentioned in the latter study, had a similar, slightly acidic pH as soil L.

A similar pattern as for *Thelebolus* was found for basidiomycetous yeasts and dimorphic fungi in the class *Tremellomycetes* (Fig. 5). Relative abundances of these two groups show a linear correlation across all samples (r = 0.72, p = 0.035). A similar correlation between certain basidiomycetous yeast and *Thelebolus* was also found by Orgiazzi et al. (2012). It will be interesting to see whether they simply have preference for similar ecological niches or a closer interdependence of a yet unknown mechanism. Many species in the *Tremellomycetes* were described as acidotolerant and heavy metal tolerant (e.g. Gadanho and Sampaio 2009) and seem to prefer lower temperatures (e.g. Wuczkowski et al. 2011). Furthermore, single isolates of cryptococcal yeasts are often described as plant growth-promoting yeasts (e.g. Liu et al. 2016), but an in-depth study of this topic is still missing.

A significant reduction in response to Cu was observed only for the highly abundant order of Hypocreales (Fig. 4). Among the Hypocreales the potentially root pathogenic genera *Dactylonectria* and *Ilyonectria* (OTU_7, OTU_9, OTU_21) were highly abundant in soil L, but nearly absent in soil D and in L5000. Species in *Dactylonectria* and *Ilyonectria* can cause black-foot disease in grapevines (Agustí-Brisach and Armengol 2013) and were described to be sensitive to copper oxychloride (Alaniz et al. 2011). Reduction in black-foot disease could thus be a beneficial side effect of Cu application in viticultural soils, but infection with this pathogen complex often already occurs in nurseries (Reis et al. 2013).

*Dactylonectria* was very sensitive to Cu application, as indicated by the EC_50_ value of 6.6 mg kg^−1^ EDTA-Cu. *Fusarium* species are generally known as plant pathogens with a high saprotrophic potential. Among the OTUs belonging to the genus of *Fusarium*, which also showed a strong dose response relationship with Cu, there are species such as *Fusarium oxysporum* and *Fursarium solani* that are well known to cause root rot. Their decline with Cu application is a beneficial non-target effect in soil. In addition, also *Fusarium graminearium*, can have negative effects in agriculture due to its mycotoxin production.

*Plectosphaerella* sp., a genus of opportunistic plant pathogenic species often found on cucurbits (Carlucci et al. 2012), became more dominant in soil D at higher Cu concentrations, which explains separation of samples D1500 and D5000 from the samples with lower Cu concentrations in PCoA (Fig. 3).

Glomeromycota were not detected in the whole dataset. The finding from other studies, where arbuscular mycorrhizal fungi where shown to be especially sensitive to heavy metal pollution could therefore not be tested in this study. Targeted studies involving specific primers targeting Glomeromycota in soil and root samples (Senés-Guerrero and Schüßler 2016) combined with traditional microscopic and spore washing techniques are needed to address this topic in more detail.

## Summary and conclusions

Strong negative effects of Cu application on the total abundance of soil microbial biomass and the fungal biomass proxy ergosterol were detected already at relatively low concentrations. Microbial respiration was reduced by Cu only in the acidic soil L. By contrast, oxidative enzyme activities were elevated in response to high levels of Cu application in the short term; in the medium term, peroxidase was strongly related to plant and root biomass. A more detailed picture was obtained by fungal community analysis. The Shannon diversity of the fungal community was mostly unaffected, but a remarkable Cu-induced change in the community composition was found, which depended on the soil properties and, hence, on Cu availability. A high number of diverse fungi were able to thrive under conditions of high Cu concentrations, whereas potentially root-pathogenic genera were strongly reduced at increasing soil Cu levels. The latter is a beneficial side effect, which can reduce black-foot disease in grapevine. Our study demonstrates that Cu additions to vineyard soils may disrupt microbial equilibria especially in less well buffered, acidic, sandy soils. However, we did not find a clear-cut decline in microbial/fungal indicators; rather, the responses were diverse with certain organisms being suppressed and others taking advantage of the temporarily extreme conditions.

## Electronic supplementary material

Electronic Supplementary Material

Online Resource_Fungal Community Data
