# Supplementary material for: Assessment of Cu applications in two contrasting soils—effects on soil microbial activity and the fungal community structure
Source: Ecotoxicology. 2018 Jan 2;27(2):217–33. doi: 10.1007/s10646-017-1888-y (PMC5847031; doi:10.1007/s10646-017-1888-y)
Supplement: Supplementary file 1 — Electronic Supplementary Material [file 10646_2017_1888_MOESM1_ESM.pdf]

Electronic Supplementary Material

Titel: "**Assessment of a Cu applications in two contrasting soil – effects on soil microbial activity and the fungal community structure**"

Journal: Ecotoxicology

Authors: Katharina M. Keiblinger[1*], Martin Schneider[1,2,#], Markus Gorfer[3,#], Melanie Paumann[1], Evi Deltedesco[1,3], Harald Berger[4], Lisa Jöchlinger[1], Axel Mentler[1], Sophie Zechmeister-Boltenstern[1], Gerhard Soja[2], Franz Zehetner[1]

Corresponding: Katharina Keiblinger, Institute for Soil Research, University of Natural Resources and Life Sciences (BOKU), Peter Jordanstrasse 82, 1190 Vienna, Austria; email: katharina.keiblinger@boku.ac.at; Tel. +43-1-47654-91141, Fax: +43 1 47654 91109

Supplementary Tables

Supplementary Table S1 Trace metal contents of the fungicide.

	mean [mg kg^{-1}]		SEM	n
Cu	334620.64			1
Mn	23.25	±	2.07	2
Fe	67.30	±	55.75	2
Ni	31.75	±	12.86	5
Zn	136.50	±	44.16	2
Sr	12.61	±	3.77	4
Pb	7.87	±	1.91	4

[a] SEM - standard error of mean

Supplementary Table S2 Basic characterization of the fungicide.

**Parameter**	**mean**
C_{org}	6.38%
N	0.3%
C/N	21.5
pH	6.3
EC	26.3 mS
Ash content	22.25%

Supplementary Table S3 Primer sequences for amplification of fungal DNA.

**ITS3-Mix**	
ITS31_NeXTf	TCGTCGGCAGCGTCAGATGTGTATAAGAGACAG**CATCGATGAAGAACGCAG**
ITS32_NeXTf	TCGTCGGCAGCGTCAGATGTGTATAAGAGACAG**CAACGATGAAGAACGCAG**
ITS33_NeXTf	TCGTCGGCAGCGTCAGATGTGTATAAGAGACAG**CACCGATGAAGAACGCAG**
ITS34_NeXTf	TCGTCGGCAGCGTCAGATGTGTATAAGAGACAG**CATCGATGAAGAACGTAG**
ITS35_NeXTf	TCGTCGGCAGCGTCAGATGTGTATAAGAGACAG**CATCGATGAAGAACGTGG**
**ITS4-Mix**	
ITS4_NeXTr	GTCTCGTGGGCTCGGAGATGTGTATAAGAGACAG**TCCTCCGCTTATTGATATGC**
ITS43S_NeXTr	GTCTCGTGGGCTCGGAGATGTGTATAAGAGACAG**TCCTSSSCTTATTGATATGC**

Primers contain fungal specific sequences at the 3' end (highlighted in bold) and a 5'-tail for sequencing at the Illumina MiSeq platform. Primers target the ITS2 region and are composed of five different forward primers – ITS31-ITS35 as specified by (Tedersoo et al., 2014) – and two different reverse primers – ITS4 (White et al., 1990) and ITS43S, a degenerate version of ITS4. Forward and reverse primers were mixed in equimolar ratios to obtain ITS3-Mix and ITS4-Mix.

Supplementary Table S4 Dose Response Relationship of microbial biomass (SMBC and Ergosterol) fitted with Sigma Plot using logistic curve fit with four parameters. EC_{50} values are given in mg Cu kg^{-1} dw, min and max of SMBC and Ergosterol are also given in mg kg^{-1} dw.

		**SMBC**		**Ergosterol**	
		**soil D**	**soil L**	**soil D**	**soil L**
**1st sampling**	EC_{50}	76	142	49	9.2
	min	80	80	0.30	1.08
	max	161	132	10.4	15.4
	R^2	0.99	0.92	0.98	0.99
**2nd sampling**	EC_{50}	84	80	94	57
	min	85	88	4.32	1.60.
	max	137	153	4.78	2.00
	R^2	0.92	0.85	0.99	0.76

Supplementary Table S5 Dose Response Relationship of microbial activity (Respiration rate and Peroxidase as well as Phenoloxidase activity) for soil L at the second sampling, fitted with Sigma Plot using logistic curve fit with four parameters. EC_{50} values are given in mg Cu kg^{-1} dw, min and max of Respiration are given in µg g^{-1} h^{-1}, while min and max of Peroxidase and Phenoloxidase activity are given in nmol g^{-1} h^{-1}.

	**Respiration**	**Peroxidase**	**Phenoloxidase**
EC_{50}	188	265	37
min	0.39	3268	6151
max	1.15	9753	9651
R^2	0.86	0.99	0.88

Supplementary Table S6 Pearson's product-moment correlation coefficients (r) for the measured parameters at the **second sampling** in both soils, within soil L and within soil D. Bold numbers indicate significant correlations with p-values below 0.001. Bold italic numbers indicate significant correlations with p-values below 0.05.

Both	*EDTA-Cu*	*pH*	*SMBC*	*Respiration*	*qCO_2*	*Peroxidase*	*Phenoloxidase*
pH	0.255	1.000					
SMBC	***-0.587***	-0.380	1.000				
Respiration	-0.255	***0.774***	0.053	1.000			
qCO_2	0.058	***0.748***	-0.451	***0.800***	1.000		
Peroxidase	-0.491	***-0.846***	***0.640***	-0.426	***-0.600***	1.000	
Phenoloxidase	0.049	***-0.663***	0.244	-0.356	-0.251	***0.706***	1.000
shoot DW	***-0.556***	0.473	0.205	***0.745***	0.473	-0.178	-0.416
root DW	***-0.574***	0.464	0.328	***0.731***	0.351	-0.180	-0.496

L	*EDTA-Cu*	*pH*	*SMBC*	*Respiration*	*qCO_2*	*Peroxidase*	*Phenol-oxidase*
pH	***0.818***	1.000					
SMBC	-0.696	-0.736	1.000				
Respiration	***-0.826***	***-0.900***	***0.846***	1.000			
qCO_2	-0.615	-0.455	0.118	0.516	1.000		
Peroxidase	***-0.759***	-0.754	***0.814***	***0.925***	0.380	1.000	
Phenoloxidase	-0.448	-0.727	***0.755***	***0.826***	0.038	***0.833***	1.000
shoot DW	-0.606	-0.706	***0.944***	***0.893***	0.243	***0.857***	***0.826***
root DW	-0.593	-0.754	***0.930***	***0.908***	0.236	***0.837***	***0.866***

D	*EDTA-Cu*	*pH*	*SMBC*	*Respiration*	*qCO_2*	*Peroxidase*	*Phenoloxidase*
pH	***0.863***	1.000					
SMBC	-0.464	-0.616	1.000				
Respiration	-0.245	0.196	-0.354	1.000			
qCO_2	0.209	0.570	***-0.822***	***0.809***	1.000		
Peroxidase	-0.684	***-0.836***	0.517	-0.385	-0.578	1.000	
Phenoloxidase	0.740	***0.770***	***-0.875***	0.228	0.709	-0.606	1.000
shoot DW	***-0.893***	***-0.805***	0.176	0.322	0.007	0.571	-0.466
root DW	***-0.945***	***-0.906***	0.515	0.203	-0.271	0.608	-0.727

Supplementary Table S7 Dose Response Relationship of the relative Abundance of fungal genera (Acremonium, Dactylonectria and Fusarium) for soil L at the second sampling, fitted with Sigma Plot using logistic curve fit with four parameters. EC_{50} values are given in mg Cu kg^{-1} dw, min and max are relative Abundance in %.

	**Acremonium**	**Dactylonectria**	**Fusarium**
EC_{50}	3.5	6.6	55
min	0.23	0.265	2.61
max	4.08	9.94	9.76
R^2	0.98	0.85	0.69

Supplementary Figures

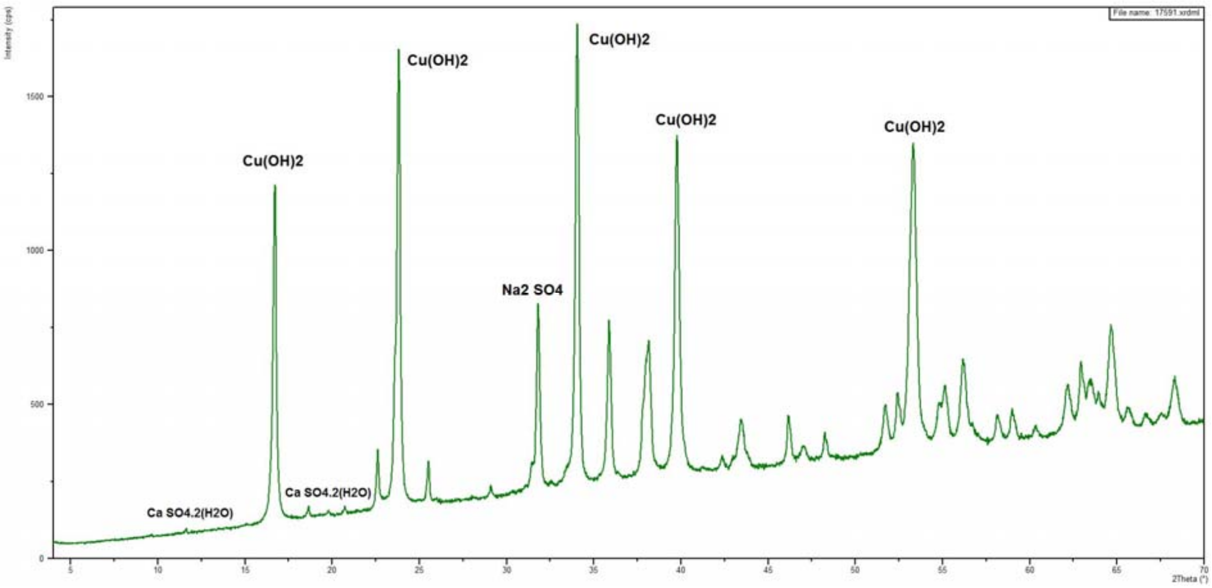

Supplementary Fig. S1 XRD results of the fungicide powder.

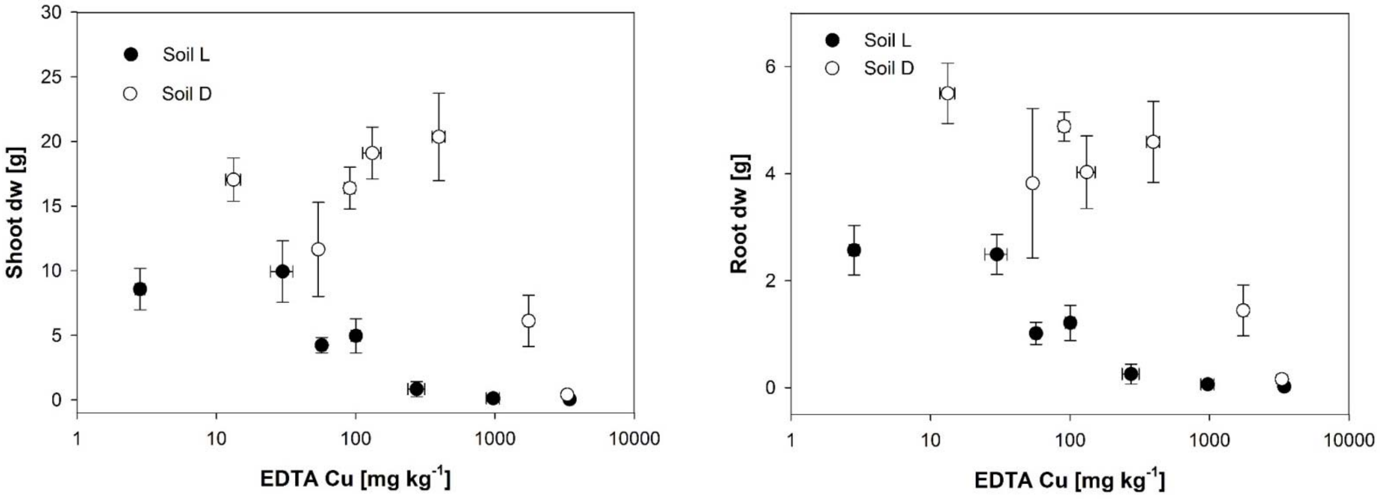

Supplementary Fig. S2 Shoot biomass [g] (left) and root biomass [g] (right) in the two different soils at the 2nd sampling in dependence of EDTA-extractable Cu. Error bars indicate standard error; n=5.

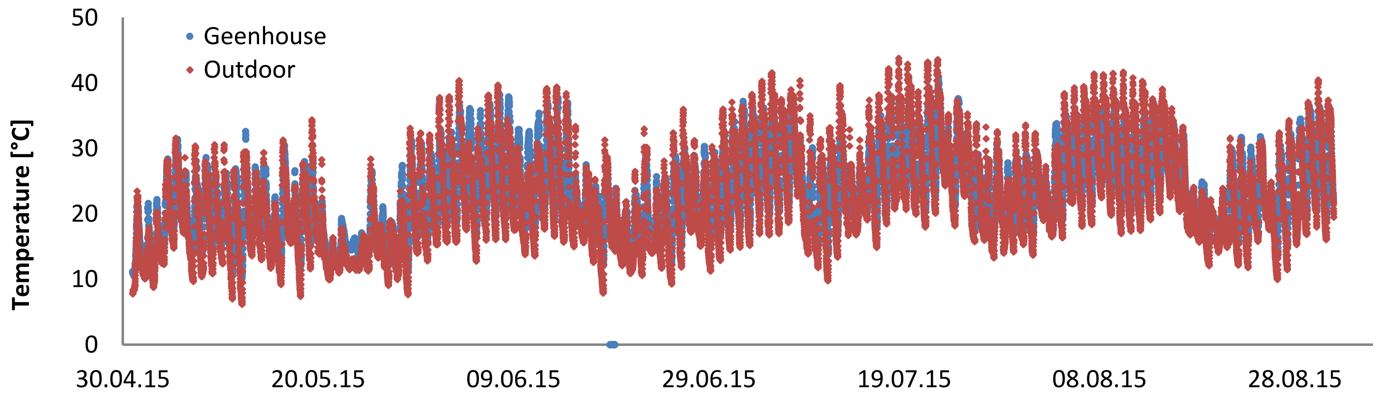

Supplementary Fig. S1 Temperature profile in the greenhouse and outdoors, over the course of incubation

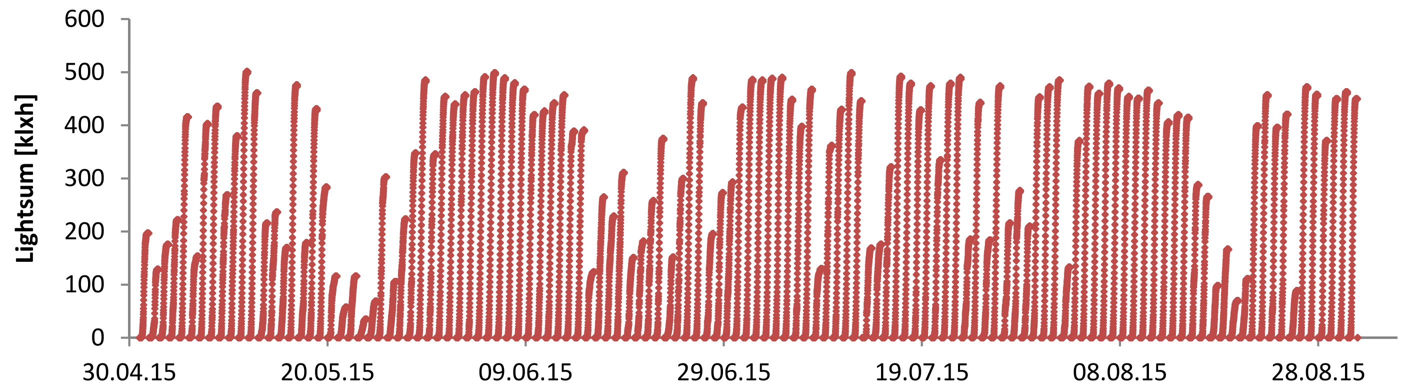

Supplementary Fig. S2 Lightsum profile in the greenhouse over the course of incubation
